# Nurses’ perceptions of metaparadigmatic concepts in anthroposophic nursing

**DOI:** 10.1590/0034-7167-2024-0596

**Published:** 2025-12-08

**Authors:** Susana Martín Hernández, Janaina Meirelles Sousa, Georgina Casanova Garrigos

**Affiliations:** I Universitat Rovira i Virgili. Facultat d’Infermeria. Tarragona, Spain; II Universidade de Brasília. Ceilândia, Distrito Federal, Brazil

**Keywords:** Nursing Care, Nursing, Anthroposophy, Nursing Theory, Complementary Therapies., Atención de Enfermería, Enfermería, Medicina Antroposófica, Teoría de Enfermería, Terapias Complementarias.

## Abstract

**Objectives:**

to identify nurses’ perceptions of the metaparadigmatic concepts emerging from nursing practice as expanded by the anthroposophic medical system.

**Methods:**

a qualitative study using semistructured interviews with 23 nurses of different nationalities about anthroposophic nursing practice. Data were analyzed through Reflective Thematic Analysis supported by ATLAS.ti^®^ software.

**Results:**

the human being is perceived as both fourfold and threefold (body, soul, and spirit), requiring care to evolve throughout one’s biography. Health is understood as a balance among bodies and systems, influenced by spirituality and the perception of biographical coherence. Nursing provides extended care through external applications, nurses’ self-knowledge, and the establishment of therapeutic and spiritual bonds.

**Final Considerations:**

the findings revealed a therapeutic approach within nursing care, expanded by the anthroposophic medical system and compatible with conventional nursing practices, indicating theoretical elements and framework that can contribute to the development of an anthroposophic nursing theory.

## RESUMEN

**Objetivos:**

identificar la percepción de enfermeros sobre conceptos metaparadigmáticos que emergen de la práctica de la enfermería ampliada por el sistema médico antroposófico.

**Métodos:**

estudio cualitativo con entrevistas semiestructuradas a 23 enfermeros de distintas nacionalidades sobre su experiencia en enfermería antroposófica. Los datos fueron analizados mediante Análisis Temático Reflexivo con apoyo del programa ATLAS.ti^®^.

**Resultados:**

el ser humano se concibe como cuatrimembrado y trimembrado (cuerpo, alma y espíritu), requiriendo cuidados para desarrollarse en su biografía. La salud se entiende como equilibrio entre cuerpos y sistemas, influida por la espiritualidad y la percepción de coherencia biográfica. La enfermería brinda cuidado ampliado mediante aplicaciones externas, autoconocimiento del profesional y vínculo terapéutico-espiritual.

**Consideraciones Finales:**

los datos evidencian una terapéutica aplicada al cuidado ampliado por el sistema médico antroposófico, compatible con la práctica de enfermería convencional, señalando elementos y referentes teóricos que respaldan la creación de una teoría de enfermería antroposófica.

## INTRODUCTION

In the discipline of nursing, the metaparadigm is understood as a set of concepts and consensus statements that support nursing knowledge and practice. Initially related to person, environment, health, and nursing, its scope has recently expanded to include other forms of knowledge, cultures, and human contexts, such as planetary health and the global environment^(1,2)^.

The theoretical and philosophical foundations of nursing provide an ontological basis and contribute to advancing the discipline; however, there is a growing desire for these theoretical assumptions to also be applied in practice^(3-6)^. The nursing metaparadigm enables professionals to think as nurses, granting them autonomy in practice, a sense of identity within a collective, and skills for interdisciplinary collaboration^(7-10)^.

Theoretical concepts in nursing adapt, shift, and evolve, just as patients’ needs change-reflected in the increasing demand for integrative care services. In Brazil, complementary and alternative medicine (CAM) was introduced into the public health system in 2006. This scenario extends to 14 of the 36 countries in the Americas, of which 8 have technical units within their Ministries of Health dedicated to CAM: Brazil, Chile, Cuba, Mexico, Nicaragua, Peru, Uruguay, and Ecuador^(11,12)^. Several factors contribute to this trend, including growing awareness of the interconnection between physical, mental, and emotional health, and the rising prevalence of chronic diseases^(13,14)^.

Current care recipients seek not only the management of diseases and disorders, but also meaning in the healing process. There is a societal trend toward reduced materialism and a growing need for the professional integration of conventional medicine with traditional and complementary medical systems as a whole^(15,16)^.

Since 1923, anthroposophic nursing (AN) has been part of the integrative care offered within the anthroposophic medical system (AMS). This system is understood as an integrative medical model practiced by physicians, therapists, and nurses, which includes, in addition to biomedical treatments, other specific therapies such as medicinal products, art therapy, movement therapy, rhythmical massage, and nursing-specific practices. AN is provided in hospitals, primary care centers, and specialized care settings^(17,18)^.

Knowledge of anthroposophic medicine has been present in European universities since 1995, with the establishment of research chairs, training programs, and projects focused on anthroposophic treatment modalities^(18)^. In 2023, the World Health Organization (WHO) published criteria for anthroposophic nursing training courses, and more recently, AN was officially recognized as a nursing specialty by the European Specialist Nurses Organisation (ESNO). In Brazil, the same recognition was granted in 2024 by the Federal Nursing Council^(19,20)^.

In Brazil, there are no hospitals guided by anthroposophic philosophy. The challenges involved in implementing AN practices in non-anthroposophic healthcare settings include the need to train nursing professionals in these therapies, raise awareness among healthcare managers about their positive effects on users’ health and quality of life, and develop research that legitimizes their relevance within the national health landscape^(21)^.

In the literature, nurses who provide anthroposophic care identify a growing movement aligned with broader societal shifts toward new models of care-aimed at finding meaning and improving disease management^(11)^. Over the past three years, studies and reviews have explored the reach of AN in addressing a variety of symptoms and health conditions. One recent study involving adults with disabilities also demonstrated the benefits of AN for behavioral regulation^(22-24)^. Another study described the use of the 12 Gestures of Care as a tool for nursing care^(25)^. The literature presents evidence of the effectiveness of anthroposophic medicine and nursing, particularly regarding improvements in quality of life, well-being, symptom relief, and treatment outcomes^(26,27)^.

This study is grounded in the anthroposophic view of the world and of the human being. Developed by Rudolf Steiner between 1886 and 1925, anthroposophy proposes a way of observing and understanding the world and the human being based on the premise that spiritual reality can be perceived through knowledge. According to this view, the human being is composed of four bodies: the physical body, consisting of matter and subject to the laws of physics and chemistry; the etheric body, related to life forces; the astral body, responsible for emotions, reactions, and sensations; and the I organization, which provides self-awareness and consciousness. Anthroposophy provides the philosophical foundations for various fields, such as Waldorf pedagogy, biodynamic agriculture, and the anthroposophic medical system^(17-19,28)^.

The development of metaparadigms open to new physical, emotional, and spiritual realities may offer nurses the opportunity to contribute to the quality of life of those under their care. This study assumes that AN, since its inception more than a century ago, has been grounded in scientific principles that support the development of a nursing theory.

## OBJECTIVES

To identify nurses’ perceptions of the metaparadigmatic concepts emerging from nursing practice as expanded by the anthroposophic medical system.

## METHODS

### Ethical considerations

The study was conducted in accordance with national and international ethical guidelines and was approved by the Research Ethics Committee of the Universitat Rovira i Virgili, Spain. Informed consent was obtained from all individuals involved in the study through an Informed Consent Form (ICF), provided in both print and online formats. Confidentiality and anonymity were ensured by assigning each nurse participant the prefix “NUR”, followed by a number indicating the order of the interview (NUR-1, NUR-2, etc.).

### Study design

This is a descriptive, qualitative study conducted through semistructured interviews focused on metaparadigmatic concepts in anthroposophic nursing. The study followed the Consolidated criteria for reporting qualitative research (COREQ) from the EQUATOR Network^(29)^.

### Study setting

Meetings between researchers and participants were scheduled in advance by phone or email. Initial contacts were provided by the head nurse of an anthroposophic institution in Germany, and subsequent participants were recruited using snowball sampling^(30)^. Participants were from Germany, Brazil, Switzerland, Chile, and Spain.

### Data source

Inclusion criteria were as follows: being a nurse; having some form of training in anthroposophic external applications and basic anthroposophic principles; working in healthcare settings (hospitals, clinics, primary healthcare units, or home care) based on anthroposophic concepts and external applications; having at least one year of experience with external applications; and being able to communicate in Portuguese, English, Spanish, or German. The sample consisted of 23 professionals who expressed interest and agreed to participate in the study.

### Data collection and organization

Data were collected between October 2023 and July 2024 through interviews conducted using a semistructured guide in Portuguese, English, Spanish, and German. Prior to data collection, the interview guide was reviewed by a panel of three nurses with expertise in anthroposophic nursing and tested in two pilot interviews to confirm its applicability.

The data collection instrument included 3 questions on sociodemographic characteristics, 8 on professional background and workplace, 4 on management, teaching, and research in anthroposophic nursing, 10 on knowledge of anthroposophy applied to health, 3 on the nurse’s personal development and self-knowledge, and 13 on anthroposophic nursing care. Interviews were conducted in a virtual meeting room, attended exclusively by the participant and the researcher, and lasted between 50 and 120 minutes. All interviews were audio taped with the participant’s consent. The researchers are native speakers of Portuguese and Spanish and are fluent in English and German.

### Data analysis

The participants’ responses were analyzed using the operational approach of Reflective Thematic Analysis (RTA), as proposed by Virginia Braun and Victoria Clarke. These authors define RTA as a flexible method for exploring qualitative data aimed at identifying, interpreting, and analyzing patterns through interpretive observation and deep immersion in the subject matter. They propose six phases for conducting the analysis:

- Familiarization with the data: reading the entire dataset and initiating the process of identifying and interpreting meanings to detect relevant phenomena and elements;
- Generating initial codes: coding features that convey meaning in the data and identifying patterns that will subsequently give rise to themes;
- Constructing themes: grouping codes that share unifying characteristics or represent a coherent and meaningful pattern in the data into potential themes;
- Reviewing initial themes: assessing the coherence and relevance of the themes in relation to the coded extracts and the entire dataset;
- Defining and naming themes: performing a preliminary thematic analysis and examining the specificities of each theme, verifying their alignment with the research questions and the phenomena under investigation, and generating clear definitions and names for each one;
- Producing the report: conducting the final analysis and describing the interpretive narrative of the data, including excerpts that contextualize the codes and themes^(31 33)^.

All interviews were first transcribed in the participant’s original language. They were then translated into Spanish and compiled in a Word document for analysis using ATLAS.ti software, Version 24. Next, the researchers selected meaningful excerpts and assigned identifying codes, which were subsequently organized into four thematic areas.

## RESULTS

All interviewed nurses were female. Most were over 50 years old, followed by a group aged between 40 and 50, and a minority between 30 and 40. Participants were equally distributed among German-speaking countries (Switzerland and Germany), Spanish-speaking countries (Chile and Spain), and a Portuguese-speaking country (Brazil), with a small number from an English-speaking country (the United States). Most had advanced training in AN, some had basic training, and a few were completing their basic AN course. All participants had experience in the clinical practice of AN; some were also involved in AN education, and a small group had experience in anthroposophic hospital management. Their areas of practice included hospital care, private practice, outpatient care, and services for people with disabilities.

For the participants, the Human Being (Chart 1) comprises multiple corporeal dimensions (fourfold and threefold constitution), consisting of body, soul, and spirit. They described the human being as a spiritually incarnated entity, capable of resilience and choice, of loving and committing to other human beings, to the environment, and to the cosmos. This being develops their life narrative with inner coherence and ethical integrity, but requires care in order to awaken a healing impulse.

**Chart 1:** Codes and verbatim transcriptions on the concept of the human being

Concept of HUMAN BEING		
**Codes**	**NUR**	**Verbatim transcriptions**
Fourfold constitution	NUR-08	“...not only do I pay attention to physical and biological processes, but I also pay attention to vital forces-how the person is emotionally-and also to what needs may exist on a spiritual level...”
Threefold corporeality	NUR-14	“...from the perspective of the physical, emotional, and spiritual approach...”
Threefold human being	NUR-10	“...One can begin from the fourfold or threefold human being and, from there, consider how these different bodies that constitute the human being are functioning, and provide the necessary care...”
Need for care	NUR-09	“...A human being who needs care in order to activate a healing impulse...”
	NUR-21	“...It depends on the anamnesis. When I talk to the patient, I understand what their needs are, where there is an imbalance, where there is a need...”
Incarnated spiritual being	NUR-12	“...being a spiritual being undergoing a terrestrial experience...”
Multidimensional resilience	NUR-12	“...the ability we have to recover. It’s about our capacity to reclaim an experience that was not fully processed on an emotional level during life...”
Capacity for decision-making	NUR-11	“...He is a being who needs care, he needs to be cared for...”
Ability to love and commit	NUR-05	“...The person lives in this state of powerlessness to love and to engage with others...”
Inner impulse, will to heal	NUR-12	“...There is something beyond my practical capabilities that relates to the other person’s determination and will to follow their own healing path...”
Connection with the environment and the cosmos	NUR-06	“...seeing people as being connected to the environment and to the cosmos as well...”
	NUR-14	“...the human being is very cold, I would say-the demands of today’s society, the materialistic way of life, are placing great pressure on the human being...”
Bearer of a life narrative and destiny	NUR-06	“...so, biographical aspects, for example, aspects of destiny...”
	NUR-04	“...(illness)... being able to make sense of it within that person’s life story-within their own biography...”

*NUR - participating nurse.*

Regarding the Environment (Chart 2), the interviewees valued ethnic, economic, dietary, and family aspects; the physical surroundings in which the person is embedded and lives; the existence of policies that allow freedom of choice in healthcare services; and the level of social trust in complementary or integrative approaches. They also noted that AN can be practiced in all healthcare settings-from pregnancy to end-of-life care-across all levels of healthcare and clinical specialties.

**Chart 2:** Codes and verbatim transcriptions on the concept of environment

Concept of ENVIRONMENT		
**Codes**	**NUR**	**Verbatim transcriptions**
All levels of care and specialties	NUR-13	“…nursing appeals to all levels of care currently recognized in the field of medicine, promoting, preventing, healing, rehabilitating, and providing follow-up…”
	NUR-11	“…it may occur in a school health context, or in emergency situations…”
	NUR-07	“…it can also provide support in cases of psychiatric illness, for example…”
Ethnic aspects	NUR-09	“Regarding ethnicity, there are cultural habits that people from different ethnic backgrounds have and, at times, they do not tolerate or accept other types of care…”
The economic factor	NUR-17	“I believe that the processes that most influence are the economic ones…”
The family	NUR-15	“…it strongly affects the family situation, the type of family that is shaped within that environment…”
Food	NUR-11	“…we’re talking about the foods people consume…”
The environment in which one lives	NUR-11	“…based on what I hear, due to the contamination of a hostile environment, a cold environment, a warlike environment…”
	NUR-16	“It’s very important to consider the person’s environment-where they live-because their inner attitudes reflect how they perceive the world…”
Trust in natural methods	NUR-05	“There’s mistrust regarding natural healing methods…”
	NUR-06	“…as well as scientific acceptance. I believe this is a very important point from my perspective. I’m not referring to public acceptance in general…”
Freedom to choose healthcare service	NUR-19	“…it has to do with the issue of choosing a healthcare service…”
	NUR-15	“…exclusive access to public healthcare services…”

*NUR - participating nurse.*

According to the participants, Health (Chart 3) is an individual process-an equilibrium between the different bodily dimensions and systems in the face of life experiences, illness, and the dying process. It is influenced by family, social, cultural, and spiritual factors, as well as by a sense of inner coherence when the individual chooses processes that promote health and the development of their own life narrative.

**Chart 3:** Codes and verbatim transcriptions on the concept of health

Concept of HEALTH		
**Codes**	**NUR**	**Verbatim transcriptions**
Balance	NUR-07	“…to find a balance between the so-called degenerative forces or formative forces.”
	NUR-06	“Then this balance collapses, and there are restrictions on one or more of these levels-physical, mental, and spiritual.”
	NUR-17	“…I have two poles-hardening and dissolution. If you look at it from this perspective, if one of these poles becomes exacerbated, it will lead to illness…”
	NUR-08	“…health and illness are on a continuum…”
	NUR-15	“…the health and illness process is based on this triad of physical body, mind, and spirit…”
Salutogenesis	NUR-18	“We’re talking about salutogenesis, which refers to the potential for health that exists within people.”
Biography	NUR-22	“I believe that age and life experience influence one’s relationship with illness.”
	NUR-09	“…you carry an unresolved biographical issue, something from childhood that hasn’t been worked through, a trauma that ends up destabilizing your health…”
Inner coherence	NUR-11	“…I don’t want to become ill, because I’m not doing something that brings me joy, something I truly want to do…”
	NUR-08	“…illness is an opportunity for inner development…”
Influence of family, culture, and society	NUR-07	“Yes, all these aspects-especially family, social, and cultural-affect the health process. (…) Classical examples include overstimulation caused by the many means of communication that lead to physical tension, difficulty concentrating, and nervousness.”
Individual	NUR-08	“…health is an individual matter-not everyone exposed to the same germ becomes ill…”
Includes spirituality	NUR-05	“…someone who crosses the threshold. They may be ill, but there’s always this perspective that something is in development, and something is still possible at this stage.”

*NUR - participating nurse.*

The participants described Nursing (Chart 4) as a practice that takes place in all healthcare contexts and specialties, grounded in an individualized, holistic, and expanded perspective of the human being, expressed through external applications. This practice aligns with both technological advancements and the use of natural substances that provide warmth and stimulate vital forces, empower patients, and balance the various bodily dimensions. They recognized the spiritual world as a reality that supports, inspires, and offers the patient a channel for healing through a respectful, therapeutic, and spiritual bond rooted in love for others. They also emphasized that self-awareness and collective reflection enable them to take the necessary actions to carry out their work effectively.

**Chart 4:** Codes and verbatim transcriptions on the concept of nursing

Concept of NURSING		
Code	NUR	Verbatim transcriptions
Integral care	NUR-22	“I would define anthroposophic nursing as care for the body, soul, and spirit.”
Expanded perspective	NUR-08	“…understanding the human being in their entirety across four levels-the physical body, the vital force level, the spiritual level, and the soul level.”
	NUR-05	“I have the feeling that anthroposophic care expands the perspective of conventional care by deepening our understanding of individuals.”
	NUR-15	“…it is understood as holistic, comprehensive care for the human being. We don’t look at body parts to treat; we look at the individual as a whole…”
Working with a salutogenic approach	NUR-04	“…I feel more focused on what would be considered salutogenesis…”
Individualized care	NUR-17	“…the anthroposophic nurse must be aware of this individuality…”
Self-awareness, consciousness, self-reflection, and coherence	NUR-07	“…the caregiver has the task of working on themselves in a self-reflective way…”
	NUR-17	“…above all, I believe it’s an inner attitude…”
	NUR-07	“…my thinking is clear, my will is, so to speak, good and aligned with the patient’s current needs, and my emotions and feelings are appropriate…”
Performs external applications	NUR-10	“The nurse can accompany patients from this perspective through external applications, oil rubs, and compresses…”
Stimulates vital forces	NUR-06	“…to support the vital forces and thus stimulate healing processes…”
Encourages patient empowerment	NUR-14	“…which allows the ‘I’ of this human being to be present in their healing process.”
Uses natural treatments	NUR-15	“…we use both minerals and herbal medicine; we use natural products…”
Provides warmth	NUR-09	“A nurse who can provide nursing care through warmth.”
Incorporates technology into care	NUR-15	“…without dismissing the technological advances we have today…”
Recognizes the spiritual dimension	NUR-08	“…care that recognizes spirituality as something real…”
The art of caring and a channel for healing	NUR-20	“Anthroposophic nursing embodies the art of caring.”
	NUR-21	“We are a channel for the patient’s healing…”
Development of karma, destiny, and a spiritual connection	NUR-05	“How one comes into the world and how one leaves it again, and how everything works together karmically.”
	NUR-10	“…through a spiritual bond that is formed with the patient.”
Attitude of empathy and love for others	NUR-20	“Love. Valuing the human being. That’s how I see it. Love for one another, because we go deep…”
Therapeutic care	NUR-03	“…the strength of anthroposophic nursing also allows me to truly act as a therapist, to have a therapeutic perspective…”
Respect for the patient	NUR-14	“…respecting their individual freedom at every stage of life…”
Applies to all contexts	NUR-10	“Anthroposophic nursing can be applied in any context-from newborns to pregnant women or to those nearing the threshold. No, there are no restrictions.”
	NUR-18	“It’s present in all contexts, isn’t it? Health promotion, prevention, treatment, rehabilitation, care.”

*NUR - participating nurse.*

## DISCUSSION

Anthroposophy views the human being as a free individual, developing through various bodily, mental, and spiritual relationships-with oneself, nature, culture, and the cosmos. The human being is considered an autonomous agent with dignity and responsibility who requires professional guidance to stimulate the organism’s self-healing potential^(17,18,34)^.

In anthroposophic anthropology, the fourfold human being comprises a physical body, which can be expressed numerically or quantitatively (e.g., weight, height, vital signs); a vitality or etheric body, which regulates bodily rhythms and regenerative processes such as sleep and digestion; an astral body, manifested through appetites, sympathies, antipathies, and emotions, and expressed through language and facial expressions; and, finally, the “I,” the bearer of self-awareness and individuality, expressed in one’s biography, destiny, life path, and spiritual activity^(17)^. Within this fourfold structure, three systems-the threefold human being-unfold: the nerve-sense system, which supports sensory perception and thought; the rhythmic system, involving breathing and circulation and enabling the experience of feeling; and the metabolic-limb system, which underpins willful action^(35)^.

Cases such as the treatment of broken heart syndrome (BHS) are documented in the AN literature, showing how stress factors can disrupt the balance between the constitutive aspects of the human being (e.g., imbalance between the vital body and consciousness, low vitality, general exhaustion, anxiety attacks)^(36)^. One patient was treated for four weeks with AN care involving various external applications with natural remedies, anamnesis based on anthroposophic anthropology, and the therapeutic involvement of practitioners, resulting in improved health status^(37)^.

Another study described *complex anthroposophic care* using AN in primary care for oncology patients. The interventions helped restore functional balance among the human constitutions, support autonomy, stimulate the immune system, facilitate coping with destiny, and enhance self-awareness^(38)^.

Attention to the patient’s environment is also an essential component of AN care. One case described in the literature involves a 5-year-old child with severe bilateral viral pneumonia. A collaborative approach integrating AN care, maternal involvement, and physician attention-within an interdisciplinary care environment-enabled recovery without using antibiotics^(39)^.

Anthroposophic literature emphasizes that each human being is responsible for their own life and biography. This responsibility promotes an autonomous, balanced, and coherent regulation of the organism through psycho-emotional and spiritual self-regulation. Based on this premise, AN treatments aim not only to restore a previous state of health but also to foster a new level of strength within the organism and the individual^(22,40)^. In AN care, understanding and addressing febrile processes can lead patients to greater awareness of their illness and of the therapeutic tools available to them in their healing journey. Lemon and ginger compresses, for instance, are therapeutic resources that help promote this awareness^(41)^.

The literature shows that AN care is based on a comprehensive and individualized understanding of the physical, mental, and spiritual needs of the person receiving care, aiming to provide protective and supportive care that helps restore independence and autonomy^(34)^. This approach-expressed through the *12 Gestures of Care*-enables professionals to engage their inner state, allowing for healing care while maintaining inner coherence. This presence of soul is particularly observed in end-of-life care, where a therapeutic atmosphere is created to stimulate warmth, vital forces, and independence, helping patients move toward the acceptance of death^(23,25,42)^. The participants’ perceptions confirmed that AN relies on its own body of knowledge and practices, supporting the expansion of integrative and complementary practices in nursing care settings.

### Study limitations

This study was limited by the scarcity of academic literature on AN, which made it difficult to broaden the discussion to other specific nursing contexts where it is applied. Another limitation lies in the use of the snowball sampling technique, which may have excluded professionals with expertise capable of contributing to the development of the metaparadigmatic concepts of AN.

### Contributions to the field of nursing

This study contributes to increasing the visibility of AN in academic literature and to its foundation as a complementary and integrative nursing approach, based on its own practices that expand conventional nursing. AN provides nurses with tools to enhance their professional practice, foster self-knowledge, and recognize the spiritual world. The nursing discipline must connect to the spiritual realms of the patient, the nurse herself, and the broader world. In today’s context, as patients seek alternative forms of care, nurses with a complementary and integrative perspective must take responsibility for providing human care that extends across all spheres of the human being.

## FINAL CONSIDERATIONS

Anthroposophic nursing care offers individuals opportunities for developing their destiny and spirituality. The metaparadigmatic concepts of AN revealed in this study suggest an expanded understanding of the human being, grounded in anthroposophic anthropology. The concept of environment ranges from the physical surroundings to the political dimensions that affect individuals. The concept of health is linked to the perception of balance among bodies and systems, influenced by spirituality and biographical coherence. Lastly, anthroposophic nursing is defined as a form of expanded care through external applications, nurse self-knowledge, and the establishment of a therapeutic and spiritual bond.

Further studies are needed to expand and deepen this topic. They will also help legitimize the body of knowledge on AN in both theoretical and practical contexts, paving the way for developing an anthroposophic nursing theory to support care, education, and research.

## Data Availability

The research data are available within the article.
