# Supplementary material for: Nurses’ perceptions of metaparadigmatic concepts in anthroposophic nursing
Source: Rev Bras Enferm. 2025 Dec 8;78(6):e20240596. doi: 10.1590/0034-7167-2024-0596 (PMC12707606; doi:10.1590/0034-7167-2024-0596)
Supplement: Translation

# Translation: Percepções de enfermeiros sobre metaparadigmas da enfermagem antroposófica

**DOI:** 10.1590/0034-7167-2024-0596pt

**Authors:** Susana Martín Hernández, Janaina Meirelles Sousa, Georgina Casanova Garrigos

**Affiliations:** I Universitat Rovira i Virgili. Facultat d’Infermeria. Tarragona, Espanha; II Universidade de Brasília. Ceilândia, Distrito Federal, Brasil

**Keywords:** Cuidados de Enfermagem, Enfermagem, Medicina Antroposófica, Teoria de Enfermagem, Terapias Complementares.

## Abstract

**Objetivos:**

identificar a percepção dos enfermeiros sobre os conceitos metaparadigmáticos que emergem da prática de enfermagem ampliada pelo sistema médico antroposófico.

**Métodos:**

estudo qualitativo com entrevistas semiestruturadas com 23 enfermeiros de diferentes nacionalidades sobre a prática de enfermagem antroposófica. Submeteram-se os dados à Análise Temática Reflexiva utilizando o programa ATLAS-ti^®^.

**Resultados:**

o ser humano é quadrimembrado e trimembrado (corpo, alma e espírito), necessitando de cuidados para desenvolver-se em sua biografia. A saúde é vista como um equilíbrio entre corpos e sistemas, influenciada pela espiritualidade e percepção da coerência biográfica. A enfermagem oferece um cuidado ampliado mediante aplicações externas, autoconhecimento do enfermeiro e vínculo terapêutico e espiritual.

**Considerações Finais:**

os dados evidenciaram uma terapêutica aplicada no cuidado de enfermagem ampliado pelo sistema médico antroposófico e compatível com práticas da enfermagem convencional, indicando características e referenciais teóricos que respaldam a criação de uma teoria de enfermagem antroposófica.

## INTRODUÇÃO

Na disciplina de enfermagem, o metaparadigma é entendido como um conjunto de conceitos e declarações de consenso que respaldam os saberes e a prática de enfermagem. Inicialmente relacionado à pessoa, ambiente, saúde e enfermagem, seu escopo foi ampliado, recentemente, para outros conhecimentos, culturas e contextos humanos, incluindo saúde planetária e ambiente global^(1,2)^.

O conhecimento teórico filosófico da enfermagem fornece uma base ontológica e faz avançar a disciplina; no entanto, há um desejo de que esses pressupostos teóricos também sejam aplicados à prática^(3-6)^. O metaparadigma da enfermagem permite que o profissional pense como enfermeiro, além de ter autonomia em sua prática, identidade como membro de um coletivo e habilidades no trabalho interdisciplinar^(7-10)^.

Os conceitos teóricos da disciplina de enfermagem se adaptam, mudam e evoluem, assim como os interesses dos pacientes, haja vista a crescente demanda por serviços que ofertem cuidados integrativos. No Brasil, as Práticas Integrativas e Complementares em Saúde (PICS) foram introduzidas no sistema público de saúde em 2006. Essa realidade se estende a 14 dos 36 países das Américas, dos quais 8 contam com unidades técnicas do Ministério da Saúde responsáveis pelo assunto: Brasil, Chile, Cuba, México, Nicarágua, Peru, Uruguai e Equador^(11,12)^. Vários fatores contribuem para esse fenômeno: a conscientização da interconexão entre saúde física, mental e emocional, assim como o aumento da taxa de doenças crônicas, entre outros^(13,14)^.

Os beneficiários dos cuidados atuais buscam tanto significado quanto gerenciamento de doenças e distúrbios de inúmeras maneiras. Há uma tendência social em direção a menos materialismo e uma necessidade crescente de integração profissional da medicina convencional e dos sistemas médicos tradicionais/complementares como um todo^(15,16)^.

Desde 1923, a enfermagem antroposófica (EA) é parte dos cuidados integrativos do sistema de medicina antroposófica (MA). Este é entendido como um sistema médico integrativo praticado por médicos, terapeutas e enfermeiros que oferece, além dos tratamentos considerados biomédicos, outros tratamentos e terapias específicas com medicamentos, arte, movimento, massagem e práticas específicas de enfermagem. A EA é praticada em hospitais, centros de atendimento primário e atendimento especializado^(17,18)^.

O conhecimento da medicina antroposófica está presente em universidades europeias, com cátedras de pesquisa, treinamento e projetos sobre modalidades de tratamento antroposófico desde 1995^(18)^. Em 2023, os critérios para os cursos de treinamento em EA foram estabelecidos pela Organização Mundial da Saúde (OMS); e, recentemente, a EA foi aceita como especialidade de enfermagem na *European Specialist Nurses Organisation* (ESNO) (https://www.esno.org/members.html), ocorrendo o mesmo no Brasil por meio do Conselho Federal de Enfermagem em 2024^(19,20)^.

No Brasil, não há hospitais norteados pela filosofia antroposófica. Os desafios que se apresentam ao implemento das práticas de EA em cenários de saúde não antroposóficos requerem a capacitação de profissionais de enfermagem nessas terapias, sensibilização dos gestores de serviços de saúde sobre os efeitos positivos dessas terapias na qualidade da saúde e vida dos usuários, bem como pesquisas que legitimem sua importância no panorama da saúde nacional^(21)^.

Na literatura, os enfermeiros que atuam no cuidado antroposófico observam um impulso para seguir o movimento social de adaptação às novas tendências de cuidado, na busca de significado e gerenciamento da doença^(11)^. Nos últimos três anos, foram publicadas pesquisas e revisões sobre o alcance da EA em relação à sua aplicabilidade em diferentes sintomatologias e doenças, destacando se um estudo recente em adultos com deficiências que também mostra os benefícios da EA em alterações comportamentais^(22-24)^. Outro estudo descreveu o uso dos 12 Gestos de Cuidado como ferramenta de cuidados^(25)^. A literatura apresenta evidências da eficácia da MA e da EA no tocante aos seus efeitos sobre a qualidade de vida, sensação de bem-estar, melhoria sintomatológica, efeitos dos medicamentos e remédios, entre outros^(26,27)^.

O presente estudo baseia-se na visão antroposófica do mundo e do ser humano. Desenvolvida por Rudolf Steiner entre 1886 e 1925, a antroposofia surgiu como uma forma de observar e compreender o mundo e o ser humano sob a premissa de que é possível alcançar a visão da realidade espiritual por meio do conhecimento. Para ela, o ser humano é composto de quatro corpos: o físico, que consiste na matéria pela qual o ser humano está sujeito às leis da física e da química; o etérico, que se relaciona com a vida; o astral, responsável pelas emoções, reações e sensações; e a organização do Eu, que proporciona a autopercepção e a consciência. A antroposofia fornece as bases filosóficas que fundamentam diferentes áreas, como a pedagogia Waldorf, a agricultura biodinâmica e o sistema MA^(17-19,28)^.

O desenvolvimento de metaparadigmas abertos a novas realidades físicas, emocionais e espirituais pode oferecer aos enfermeiros a oportunidade de contribuir para a qualidade de vida da pessoa cuidada. Parte-se do pressuposto de que a EA, desde seu início há mais de um século, possui os pilares de cientificidade que sustentam o desenvolvimento de uma teoria de enfermagem.

## OBJETIVOS

Identificar a percepção de enfermeiros sobre os conceitos metaparadigmáticos que emergem da prática de enfermagem ampliada pelo sistema médico antroposófico.

## MÉTODOS

### Aspectos éticos

O estudo foi conduzido de acordo com as diretrizes de ética nacionais e internacionais e aprovado pelo Comitê de Ética em Pesquisa da *Universitat Rovira i Virgili*, Espanha. Obteve-se o Consentimento Livre e Esclarecido de todos os indivíduos envolvidos no estudo por meio de um Formulário de Consentimento Informado, impresso e online. Garantiu-se a confidencialidade e o anonimato dos dados, utilizando o prefixo “INF” para identificar o enfermeiro participante, seguido de número indicando a ordem da entrevista (INF-1, INF-2…).

### Tipo de estudo

Este é um estudo descritivo, de natureza qualitativa, realizado por meio de entrevista semiestruturada sobre os metaparadigmas da enfermagem antroposófica. Seguiram-se as diretrizes do *Consolidated criteria for reporting qualitative research* (COREQ), da Rede Equator^(29)^.

### Cenário do estudo

O encontro entre os pesquisadores e os participantes foi previamente agendado por telefone ou e-mail. Inicialmente, os contatos foram fornecidos por um enfermeiro diretor de uma instituição antroposófica alemã; e, posteriormente, os potenciais participantes foram eleitos por meio da técnica bola de neve^(30)^. Os participantes eram oriundos da Alemanha, Brasil, Suíça, Chile e Espanha.

### Fonte de dados

Estes foram os critérios de inclusão: ser enfermeiro; ter algum tipo de formação em aplicações externas antroposóficas e conceitos básicos de antroposofia; atuar em contextos de saúde (hospitais, clínicas de saúde, unidades de saúde de atenção primária e domicílio) valendo-se dos conceitos da antroposofia e das aplicações externas antroposóficas; ter tempo de experiência em aplicações externas superior ou igual a um ano; expressar-se em língua portuguesa, inglesa, espanhola ou alemã. A amostra consistiu em 23 profissionais que manifestaram interesse e aceitaram participar do estudo.

### Coleta e organização dos dados

A coleta de dados ocorreu entre outubro de 2023 e julho de 2024 mediante entrevista usando um roteiro semiestruturado em língua portuguesa, inglesa, espanhola e alemã. Antes da coleta de dados, o roteiro de perguntas foi analisado por um júri de três enfermeiros com expertise em enfermagem antroposófica e testado em duas entrevistas-piloto para confirmação de sua aplicabilidade.

O instrumento de coleta de dados contava com 3 questões para caracterização sociodemográfica, 8 questões sobre carreira profissional e local de trabalho, 4 sobre gestão ensino pesquisa em enfermagem antroposófica, 10 sobre o conhecimento de antroposofia aplicada à saúde, 3 sobre o autoconhecimento-caminho pessoal do enfermeiro e 13 sobre a assistência de enfermagem antroposófica. As entrevistas foram realizadas em uma sala virtual, na presença exclusiva do participante e do pesquisador, com duração mínima de 50 minutos e máxima de 120 minutos; e foram registradas por meio de gravação de áudio, com autorização do participante. Os pesquisadores têm como língua materna o português e o espanhol, além de serem fluentes nas línguas inglesa e alemã.

### Análise dos dados

A análise dos discursos seguiu a proposta operativa da Análise Temática Reflexiva (ATR) de Virginia Braun e Victoria Clarke, que a definem como uma abordagem flexível para considerar dados qualitativos, a fim de identificar, interpretar e analisar padrões por meio de uma observação interpretativa de dados e de uma imersão profunda no tema proposto. Essas autoras sugerem a condução da análise em seis etapas:

- Familiarização inicial com os dados: leitura do conjunto de dados e início do processo de identificação e interpretação de seus significados no intuito de perceber os fenômenos e elementos que possam ser relevantes;
- Geração de códigos iniciais: codificação de características atribuidoras de significado aos dados e identificação de padrões que irão gerar posteriormente os temas;
- Construção de temas: agrupamento de códigos que partilham características unificadoras ou descrevam um padrão coerente e significativo nos dados, em temas potenciais;
- Revisão de temas iniciais: verificação da funcionalidade dos temas em relação aos extratos codificados e todo o conjunto de dados;
- Definição e nomeação de temas: análise temática inicial e das especificidades de cada tema, verificando se estão relacionados às perguntas de pesquisa e aos fenômenos por ela tratados, gerando definições e nomes claros para cada um deles;
- Produção do relatório: análise final e descrição da história interpretativa dos dados, com a inclusão de seus extratos, permitindo a contextualização dos códigos e temas^(31-33)^.

Para análise, todas as entrevistas foram primeiramente transcritas na língua original do participante. Posteriormente, foram traduzidas para o idioma espanhol e inseridas em arquivo de texto Word no software ATLAS.ti - Versão 24. Em seguida, os pesquisadores selecionaram os trechos significativos e atribuíram códigos identificadores, que posteriormente foram organizados em quatro áreas temáticas.

## RESULTADOS

As enfermeiras entrevistadas eram todas do sexo feminino. A maioria tinha mais de 50 anos, outro grupo tinha entre 40 e 50 anos, e uma minoria tinha entre 30 e 40 anos. A origem das informantes distribuiu-se equitativamente entre as de língua alemã (Suíça e Alemanha), espanhola (Chile e Espanha) e portuguesa (Brasil), havendo uma pequena amostra de língua inglesa (Estado Unidos). A maioria tinha formação especializada em EA; algumas, em formação básica; e um pequeno grupo estava concluindo o curso básico em EA. Todas as participantes tinham experiência na prática clínica de EA; algumas, também no ensino de EA; e um pequeno grupo era experiente em gestão hospitalar antroposófica. As áreas de atuação eram assistência hospitalar, consultório particular, assistência ambulatorial e atendimento a pessoas com deficiência.

Para as informantes, o Ser Humano (Quadro 1) encerra várias corporalidades (quadrimembração e trimembração), sendo portador de corpo, alma e espírito. Consideram que o ser humano é um ser espiritualmente encarnado, com capacidade de resiliência e decisão, que pode amar e se comprometer com outros seres humanos, com o meio ambiente e com o cosmos, com coerência interna e ética no desenvolvimento de sua biografia, mas que necessita de cuidados para encontrar um impulso de cura.

**Quadro 1:** Códigos e transcrições verbatim sobre o conceito de ser humano

Conceito de SER HUMANO		
**Códigos**	**INF**	**Transcrições verbatim**
Quadrimembração	INF-08	“…não apenas presto atenção aos processos físicos e biológicos, mas também presto atenção às forças vitais, como a pessoa está animicamente e também, quais necessidades podem existir em um nível espiritual…”
Tricorporalidade	INF-14	“…do ponto de vista da abordagem física, anímica e espiritual…”
Trimembração	INF-10	“…Se pode ver a partir da quadrimembração ou a partir da trimembração e aí ver como estão esses diferentes corpos que constituem o ser humano e fornecer os cuidados necessários.”
Necessidade de cuidados	INF-09	“…Um ser humano que necessita de cuidados para encontrar um impulso de cura…”
	INF-21	“…depende da anamnese. Quando converso com o paciente, percebo quais são suas necessidades, onde há um desequilíbrio, onde há uma necessidade.”
Ser espiritual encarnado	INF-12	“…é um ser espiritual vivendo uma experiência terrena…”
Resiliência multidimensional	INF-12	“…a capacidade que temos de nos recuperar. Veja bem, é a capacidade que temos de nos recuperar da experiência que nos acontece multidimensionalmente na vida.”
Capacidade de decisão	INF-11	“Ele é um ser que pode ser cuidado, se ele quiser ser cuidado.”
Capaz de amar e se comprometer	INF-05	“A pessoa vive nesse estado de impotência para amar e se envolver com os outros.”
Dispõe de impulso interno, vontade de curar	INF-12	“…há algo que vai além de minhas próprias capacidades e que tem a ver com a determinação e a vontade do outro de seguir seu próprio caminho de cura.”
Conectado ao meio ambiente e ao cosmos	INF-06	“…ver as pessoas em conexão com o meio ambiente e também com o cosmos…”
	INF-14	“…o ser humano é muito frio, eu poderia dizer, as exigências da sociedade atual, o modo de vida materialista está exigindo muito do ser humano…”
Portador de uma biografia, destino	INF-06	“então, os aspectos biográficos, por exemplo, os aspectos do destino…”
	INF-04	“(doença)… ser capaz de dar sentido a ela dentro da história de vida dessa pessoa, de sua própria biografia…”

*INF - enfermeira participante.*

Como Meio Ambiente (Quadro 2), as entrevistadas valorizaram os aspectos étnicos, econômicos, alimentares, familiares, o entorno físico em que o ser humano está inserido e vive, as políticas que possibilitam a liberdade de escolha dos serviços de saúde e a confiança social nos meios complementares ou integrativos. Consideraram também que a EA pode ser realizada em todos os contextos de saúde, desde a gestação até o acompanhamento nos últimos dias de vida, em todos os níveis de atenção à saúde e em todas as especialidades clínicas.

**Quadro 2:** Códigos e transcrições verbatim sobre o conceito de meio ambiente

Conceito de MEIO AMBIENTE		
**Códigos**	**INF**	**Transcrições verbatim**
Todos os níveis de atendimento e especialidades	INF-13	“…a enfermagem apela a todos os âmbitos em que hoje conhecemos no campo da medicina, promovendo, prevenindo, curando, reabilitando e acompanhando.”
	INF-11	“…pode ser em um contexto escolar de saúde escolar, pode ser em situações de emergência…”
	INF-07	“…também pode ser um apoio em caso de doença na psiquiatria, por exemplo.”
Aspectos étnicos	INF-09	“Em relação às etnias, há costumes que as pessoas de diferentes etnias têm e, às vezes, elas não toleram ou não aceitam outros tipos de saúde…”
O fator econômico	INF-17	“Acho que os processos que mais podem nos influenciar são os econômicos.”
A família	INF-15	“…interfere muito a questão familiar, o tipo de família que se constrói nesse ambiente…”
Alimentos	INF-11	“…estamos falando sobre os alimentos que consome”.
O ambiente em que vive	INF-11	“…pelos sons que ouço, pela contaminação de um ambiente hostil, um ambiente frio, um ambiente de guerra…”
	INF-16	“É muito importante levar em conta o ambiente da pessoa, onde ela vive, porque suas atitudes internas refletem a maneira como ela vê o mundo…”
Confiança nos métodos naturais	INF-05	“Desconfiança em relação aos métodos naturais de cura.”
	INF-06	“…e também a aceitação científica. Acho que esse é um ponto importante do meu ponto de vista. No que diz respeito à aceitação do público em geral.”
Liberdade de escolha do serviço de saúde	INF-19	“…Tem a ver com a questão de escolher um serviço de saúde.”
	INF-15	“…acesso exclusivo ao serviço público de saúde…”

*INF - enfermeira participante.*

Para as entrevistadas, a Saúde (Quadro 3) é individual, um equilíbrio entre diferentes corporalidades e sistemas diante das experiências de vida, da doença e do processo de morrer. Ela é influenciada por aspectos familiares, sociais, culturais, espirituais e pela coerência interna quando o indivíduo escolhe processos que promovem a saúde e o desenvolvimento de sua própria biografia.

**Quadro 3:** Códigos e transcrições verbatim sobre o conceito de saúde

Conceito de SAÚDE		
**Códigos**	INF^*^	**Transcrições verbatim**
Equilíbrio	INF-07	“…encontrar um equilíbrio entre as chamadas forças degradantes ou forças formativas”.
	INF-06	“Então esse equilíbrio cai, e há restrições em um ou mais desses níveis físico, mental e espiritual.”
	INF-17	“…tenho dois polos, endurecimentos e dissoluções. Se você pensar desse ponto de vista, se um desses dois polos está exacerbado, causará doenças…”.
	INF-08	“…saúde e doença são um contínuo…”
	INF-15	“…o processo de saúde e doença se baseia nessa tríade de corpo físico, mental e espiritual…”.
Salutogênese	INF-18	“Estamos falando de salutogênese, que consiste em dar conta do potencial de saúde que têm as pessoas.”
Biografia	INF-22	“Acredito que a idade e a experiência de vida de uma pessoa influenciam sua relação com a doença.”
	INF-09	“…se você tem um problema biográfico que não resolveu, que traz da infância e que não resolveu, um trauma sempre está desarmonizando sua saúde…”.
Coerência interna	INF-11	“…eu poderia ficar doente, pois não estou fazendo algo que esteja a meu gosto, com o que eu quero…”.
	INF-08	“…a doença é uma oportunidade para o desenvolvimento interior…”
Influência da família, da cultura e da sociedade	INF-07	“Sim, todos esses aspectos, especialmente os familiares, sociais e culturais, influenciam o processo de saúde. (…) Exemplos clássicos seriam a superestimulação provocada pelos numerosos meios de comunicação que conduzem à frieza física, à falta de concentração e ao nervosismo.”
Individual	INF-08	“…a saúde é uma coisa individual, nem todo mundo que é exposto ao mesmo germe contrai a doença…”
Inclui espiritualidade	INF-05	“…uma pessoa que cruza o limiar. Ela não se refere como doente, mas sempre tem este enfoque de que há algo em desenvolvimento, e a cura também é possível nesta etapa.”

*INF - enfermeira participante.*

As informantes descreveram a Enfermagem (Quadro 4) como aquela que atua em todos os contextos e especialidades de saúde, com uma visão individualizada, integral e ampliada do ser humano por meio de aplicações externas. Essa ação está alinhada aos avanços tecnológicos e às substâncias naturais, que trazem calor e estimulam as forças vitais, o empoderamento do paciente e o equilíbrio das diferentes corporalidades. Reconheceram o mundo espiritual como uma realidade que apoia, estimula e proporciona ao paciente um canal de cura mediante um vínculo respeitoso, terapêutico e espiritual de amor ao próximo. Referiram que o autoconhecimento e a reflexão do enfermeiro como um coletivo permitem a ação necessária para a realização de seu trabalho.

**Quadro 4:** Códigos e transcrições verbatim sobre o conceito de enfermagem

Conceito de ENFERMAGEM		
Código	INF	Transcrições verbatim
Cuidado integral	INF-22	“Eu definiria a enfermagem antroposófica como enfermagem para o corpo, a alma e o espírito.”
Visão ampliada	INF-08	“…compreender o ser humano na sua totalidade com estes quatro níveis do corpo físico, o nível da força vital, o nível espiritual e também o nível da alma.”
	INF-05	“Tenho a sensação de que o cuidado antroposófico amplia a perspectiva do cuidado normal através da compreensão das pessoas, através de outra compreensão ampliada das pessoas.”
	INF-15	“…se entende como um cuidado holístico, integral do ser humano, não olhamos para partes do corpo da pessoa para tratar, olhamos para o indivíduo como um todo…”
Trabalhar com uma abordagem salutogênica	INF-04	“…sinto que estou mais focada no âmbito do que seria a salutogênese…”
Atenção individualizada	INF-17	“…a enfermeira antroposófica deve estar ciente desta individualidade…”
Autoconhecimento, consciência, autorreflexão e coerência	INF-07	“…o cuidador tem a tarefa de trabalhar sobre si mesmo de uma maneira autorreflexiva…”
	INF-17	“…principalmente, acho que é uma atitude interior…”
	INF-07	“…meu pensamento é claro, minha vontade é, por assim dizer, boa e correta para as necessidades atuais do paciente e minhas emoções e sentimentos são adequados…”
Realiza aplicações externas	INF-10	“A enfermeira pode acompanhar os pacientes nesta perspectiva com aplicações externas, oleações, envoltórios…”
Estimula as forças vitais	INF-06	“…apoiar as forças vitais e, assim, estimular os processos de cura…”
Estimula o empoderamento do paciente	INF-14	“…que permite que o Eu desse ser humano esteja presente em seu processo de cura.”
Faz uso de tratamentos naturais	INF-15	“…utilizamos tanto minerais como fitoterapia, utilizamos produtos naturais…”
Fornece calor	INF-09	“Uma enfermeira que pode fornecer cuidado de enfermagem através do calor.”
Trabalha com tecnologia	INF-15	“…Sem se esquivar dos avanços que temos hoje em tecnologia…”
Reconhece o espiritual	INF-08	“…cuidados que reconhecem o espiritual como algo real…”
Arte de cuidar e canal de cura	INF-20	“A enfermagem antroposófica tem a arte de cuidar.”
	INF-21	“Somos um canal para a cura do paciente…”
Desenvolvimento de carma, destino e vínculo espiritual	INF-05	“Como chega à Terra e como deixa a Terra novamente e como tudo ‘carmicamente’ funciona em conjunto.”
	INF-10	“…através de um vínculo espiritual que ocorre com o paciente.”
Atitude de empatia e amor pelos outros	INF-20	“Amor. Valorização do ser humano. Como eu vejo isso. Amor ao próximo, porque vamos ao fundo…”
Cuidados terapêuticos	INF-03	“…o poder da enfermagem antroposófica também me permite ser realmente um terapeuta, olhar terapeuticamente…”
Respeito pelo paciente	INF-14	“…respeitando sua própria liberdade em qualquer etapa da vida…”
Aplica-se a todos os contextos	INF-10	“A enfermagem antroposófica pode ser aplicada em qualquer contexto, desde recém-nascidos a gestantes ou em seres que estão prestes a cruzar o limiar, jovens… não, não há restrição.”
	INF-18	“Está em todos os contextos, não é? Promoção, prevenção, tratamento, reabilitação, cuidados.”

*INF - enfermeira participante.*

## DISCUSSÃO

A antroposofia vê o ser humano como um ser livre, em desenvolvimento nas mais diversas relações corporais, mentais e espirituais consigo mesmo, com a natureza, com a cultura e com o cosmos. Ele é um agente autônomo com dignidade e responsabilidade, que precisa de orientação profissional para estimular o potencial de seu organismo de auto curar-se^(17,18,34)^.

Na antropologia antroposófica, o ser humano quadrimembrado é portador de um corpo físico, que pode ter uma expressão numérica ou quantitativa, como peso, altura e sinais vitais; uma vitalidade ou corpo etérico, que é o regulador dos ritmos corporais e dos processos regenerativos, como o sono e a digestão; um corpo astral, que se manifesta por meio de apetites, simpatias, antipatias, emoções e se expressa por meio da linguagem e das expressões faciais; e, finalmente, um Eu portador de autoconsciência e individualidade, que se expressa em sua biografia, destino, caminhos de vida e atividades espirituais^(17)^. Dentro dessa quadrimembração, desenvolvem-se três sistemas (trimembração), os quais direcionam os processos nervoso-sensoriais, que proporcionam a estrutura para a percepção sensorial e pensamento; os processos rítmicos, que abrangem a respiração e a circulação e fornecem a base para a percepção do sentimento; e os processos metabólicos-motores, que formam o eixo da força de vontade^(35)^.

Casos como o tratamento da síndrome do coração partido (BHS) são descritos na literatura de EA, detalhando como os fatores estressantes afetam a harmonia das corporeidades em uma paciente (desequilíbrio entre o corpo vital e a consciência, baixa vitalidade, exaustão geral e ataques de ansiedade)^(36)^. A paciente foi tratada durante quatro semanas com cuidados de EA baseados em diferentes aplicações externas com remédios naturais, anamnese baseada na antropologia antroposófica e envolvimento terapêutico dos praticantes, trazendo melhora de seu estado de saúde^(37)^.

Outro artigo descreve o “cuidado antroposófico complexo” com a EA na atenção primária em pacientes oncológicos. Os cuidados de EA ajudaram a equilibrar a regulação funcional das corporalidades dos pacientes, apoiar sua autonomia, estimular o sistema imunológico, lidar com o destino e aumentar a autoconsciência^(38)^.

Por outro lado, a atenção ao ambiente em que o paciente está inserido é importante no tratamento de EA. Na literatura, encontramos o caso de uma criança de 5 anos com pneumonia viral bilateral grave, na qual a intervenção conjunta de cuidados de EA e a atenção da mãe e dos médicos em um ambiente de trabalho com diálogo interdisciplinar conseguiram evitar o uso de antibióticos e restabelecer a saúde^(39)^.

Na literatura antroposófica, entende-se que todo ser humano é responsável por sua própria vida e biografia. Essa responsabilidade cria uma regulação autônoma, equilibrada e coerente do organismo com sua autorregulação psicoemocional e espiritual. Sob essa premissa, os tratamentos de EA não visam apenas restaurar a condição saudável anterior, mas também provocar um novo nível de força no organismo e no indivíduo^(22,40)^. Nos cuidados de EA, reconhece-se que o tratamento e a compreensão dos processos febris provocam uma mudança na consciência do paciente sobre seu processo de doença e das ferramentas que pode utilizar em seu caminho de cura. Por exemplo, as compressas de limão e gengibre são recursos que permitem aos pacientes essa conscientização^(41)^.

A literatura mostra que os cuidados de EA baseiam-se em uma compreensão integral e individualizada das necessidades físicas, mentais e espirituais da pessoa cuidada, com o objetivo de fornecer cuidados de proteção e acompanhamento para recuperar a independência e a autonomia^(34)^. Esse cuidado de EA expresso nos 12 Gestos do Cuidado proporciona ao profissional o desdobramento de seu estado anímico, possibilitando realizar o cuidado curativo e manter a coerência. Observa-se essa postura anímica nos processos com pacientes nos últimos dias de vida, em que se cria a atmosfera necessária de estimulação de calor, das forças vitais e da independência para aproximar os pacientes da aceitação de sua morte^(23,25,42)^. A percepção das entrevistadas revelou que a EA trabalha com conhecimentos e práticas próprias, corroborando a ampliação do uso de práticas integrativas e complementares em contextos de assistência de enfermagem.

### Limitações do estudo

Como limitação do estudo, encontramos a escassez de literatura acadêmica sobre EA, não sendo possível ampliar a discussão a outros contextos específicos de enfermagem em que ela se aplica. Considera-se que a busca de participantes por meio da técnica bola de neve pode ter excluído aqueles que teriam expertise para contribuir com a ampliação dos conceitos metaparadigmáticos de EA.

### Contribuições para o campo da enfermagem

Esta pesquisa contribui para a visibilidade da EA na literatura acadêmica e para a sua fundamentação enquanto enfermagem complementar e integrativa, utilizando suas próprias práticas que ampliam a enfermagem clássica. A EA fornece aos enfermeiros ferramentas para a capacitação em seu trabalho profissional, o autoconhecimento e o reconhecimento do mundo espiritual. A disciplina de enfermagem precisa vincular-se aos mundos espirituais do paciente, da própria enfermeira e do mundo em geral. No momento atual, em que o paciente está buscando outras formas de cuidado, os enfermeiros com uma visão integrativa e complementar devem tomar em suas próprias mãos o cuidado do ser humano e estendê-lo a todas as esferas.

## CONSIDERAÇÕES FINAIS

O cuidado de EA oferece ao ser humano oportunidades de desenvolvimento de seu destino e da espiritualidade. Os conceitos metaparadigmáticos sobre a EA revelados neste estudo propõem um conceito de ser humano ampliado pela antropologia antroposófica. A definição de meio ambiente considera desde o entorno físico até os aspectos políticos que envolvem o ser humano. Já o conceito de saúde vincula-se à percepção do equilíbrio entre corpos e sistemas, que são influenciados pela espiritualidade e percepção da coerência biográfica. Por fim, a enfermagem antroposófica é definida como aquela que oferece um cuidado ampliado mediante aplicações externas, autoconhecimento do enfermeiro e vínculo terapêutico e espiritual.

Estudos futuros são necessários para ampliar e aprofundar esta temática. Também servirão para legitimar o conhecimento de EA em contextos teóricos e práticos que permitam desenvolver uma teoria de enfermagem antroposófica a fim de sustentar a assistência, o ensino e a pesquisa.

## Data Availability

Os dados da pesquisa estão disponíveis no corpo do artigo.
